# Allostatic Load Biomarker Associations with Depressive Symptoms Vary among US Black and White Women and Men

**DOI:** 10.3390/healthcare6030105

**Published:** 2018-08-28

**Authors:** Ganga S. Bey, Bill M. Jesdale, Christine M. Ulbricht, Eric O. Mick, Sharina D. Person

**Affiliations:** Department of Quantitative Health Sciences, University of Massachusetts Medical School, Worcester, MA 01655, USA; bill.jesdale@umassmed.edu (B.M.J.); christine.ulbricht@umassmed.edu (C.M.U.); Eric.mick@umassmed.edu (E.O.M.); sharina.person@umassmed.edu (S.D.P.)

**Keywords:** chronic stress, allostatic load, depression, gender, race, intersectionality

## Abstract

The prevalence and severity of depression differ in women and men and across racial groups. Psychosocial factors such as chronic stress have been proposed as contributors, but causes of this variation are not fully understood. Allostatic load, a measure of the physiological burden of chronic stress, is known to be associated with depression. Using data from the National Health and Nutrition Examination Survey 2005–2010, we examined the associations of nine allostatic load biomarkers with depression among US black and white adults aged 18–64 years (*n* = 6431). Depressive symptoms were assessed using the Patient Health Questionaire-9; logistic models estimated adjusted odds of depression based on allostatic load biomarkers. High-risk levels of c-reactive protein were significantly associated with increased odds of depression among white women (adjusted odds ratio (aOR) = 1.7, 95% CI: 1.1–2.5) and men (aOR = 1.8, 95% CI: 1.1–2.8) but not black women (aOR = 0.8, 95% CI: 0.6–1.1) or men (aOR = 0.9, 95% CI: 0.5–1.5). Among black men, hypertension (aOR = 1.7, 95% CI: 1.1–2.7) and adverse serum albumin levels (aOR = 1.7, 95% CI: 1.0–2.9) predicted depression, while high total cholesterol was associated with depression among black women (aOR = 1.6, 95% CI: 1.0–2.7). The associations between allostatic load biomarkers and depression varies with gendered race, suggesting that, despite consistent symptomatology, underlying disease mechanisms may differ between these groups.

## 1. Introduction

A large proportion of the United States chronic disease burden is attributed to depressive disorders [1]. Major depressive disorder (MDD), the most common form of depression, is the leading cause of disability among those aged 15 years and older [2]. Of central public health concern are racial and gender disparities in who develops depression; differences in the prevalence and incidence of MDD diagnosis and depressive symptomatology between black and white women and men have been well documented [3,4,5,6]. Specifically, rates of MDD diagnosis are higher among white persons. Yet, black women and men report a higher prevalence of depressive symptoms. Further, there is evidence that somatic symptoms are more common among black persons, while affective symptoms of depression are more frequently reported among whites [7].

Consensus has yet to be reached on what proportion of this disparity can be attributed to true differences in disease prevalence and manifestation between these groups as opposed to cultural and social factors yielding underreporting, underdiagnosis, and undertreatment of depression in black persons and men [8]. A host of genetic and social factors are thought to be associated with the likelihood of developing depression [4,9,10,11,12,13,14], further underscoring a need for additional investigation. In concert with potential surveillance inconsistencies, persistent uncertainty about the mechanisms for these risk factors [9] render efforts to characterize and alleviate true racial and gender disparities in depression particularly challenging.

One approach to identifying mechanisms for an effect of social status on the development of depression is the use of allostatic load, a measure of the physiological wear and tear accumulating from sustained stress exposure. The concept of allostatic load within the epidemiological discipline has improved efforts to evaluate the role of race and gender inequity in yielding depression disparities [10]. Social environment theories for gender differences in depression attribute much of the increased risk for depression among women to chronic strain associated with the subordinate social position women occupy [4,11,15]. A growing body of evidence supports a number of key physiological measures as markers of chronic stress burden associated with psychosocial exposures stemming from membership in a disadvantaged social group. Adverse levels of neuroendocrine, cardiovascular, metabolic, and immunological biomarkers comprising AL have been linked to perception of social rejection, marginalization, and exclusion [11,16,17,18,19]. Specifically, this research emphasizes the role of inflammatory processes in mediating the effect of threatening social stimuli, such as identity threat stemming from perceived racial or gender discrimination, on health [11,16]. As evidence for depression as an inflammatory disease emerges [11,20,21], studies linking psychosocial risk factors with inflammatory indicators of chronic stress offer biologically plausible mechanisms for depression as a manifestation of the chronic stress associated with exposure to structural inequity.

“Paradoxical” findings of lower rates of MDD among black persons [22,23], who, like women, occupy marginalized social positions [24], complicate social environment theories. The impact of socioeconomic factors on depression varies between black and white communities [3], with socioeconomic position largely accounting for gender disparities among black persons but not white. More work is needed to understand these divergent findings in regard to the pathogenesis of depression across these groups.

In efforts to account for gender–race differences in the relationship of chronic psychosocial stress with depression, etiologic inquiry has increasingly turned to epigenetic explanations, centering the interdependency of biological and social risk factors for disease [9]. Within such sociobiological frameworks, further consideration of the psychosocial exposures individuals encounter at the junction of gender and race (“gendered racial” exposures) becomes integral to clarifying the causes of persistent differences in depression morbidity. Examined through the lens of intersectionality theory [25], intersecting axes of structured inequity on the basis of gender and race impose a set of chronic social stressors whose effects on health cannot be reasonably separated into individual racial and gendered components. Acting concomitantly with biological vulnerability, these unique gendered racial exposures may serve as catalysts for psychopathology among certain populations but not others, as proposed by the differential effect hypothesis [26], in addition to shaping the way psychological distress manifests within these groups. Research investigating the nature of social group variation in the effects of chronic strain on mental health may therefore provide valuable insight into the causes and magnitude of gendered racial differences in risk for depression and elucidate opportunities for improvement in treatment efficacy.

Although previous literature has identified an association of inflammation with depression among white but not black persons [27,28], to our knowledge no US study has examined the extent to which gender and race simultaneously moderate the association of individual allostatic load components with depression. To address this lacuna, this analysis uses a nationally representative sample to explore variation in the relationships between nine allostatic load biomarkers of chronic stress and depressive symptoms among black and white women and men.

## 2. Materials and Methods

### 2.1. Data

We used data from the National Health and Nutrition Examination Survey (NHANES), 2005–2010. These years were combined to maximize sample size; the analysis was limited to this time period because a different depression measure was used prior to 2005 and all biomarkers of interest were not included after 2010. Conducted by the National Center for Health Statistics (NCHS), NHANES uses weighted samples to provide national estimates of health and nutritional status for the noninstitutionalized population of the United States. Study staff use specially designed and equipped mobile health centers that travel to locations throughout the country to take health measurements on about 5000 participants in 15 counties annually. NHANES data collection methodology has been further documented elsewhere [29].

### 2.2. Participants

Our analytic sample included men and women aged 18–64 years who self-identified as non-Hispanic black or non-Hispanic white (referred to hereafter as “black” and “white”) from options provided by investigators that included Mexican-American, Other Hispanic, Non-Hispanic White, Non-Hispanic Black, and Other Race—including Multi-Racial. Pregnant women (*n* = 490) were excluded, as pregnancy can alter a number of physiological measures comprising allostatic load [30,31]. Of the 14,050 participants aged 18–64 years in NHANES 2005–2010, we further excluded participants whose reported race was not black or white (*n* = 5025), those missing information on any of the questions included in the depression measure (*n* = 1824), AL biomarkers of interest (*n* = 2530), and/or family poverty–income ratio (PIR, *n* = 1120).

### 2.3. Depressive Symptoms

Participants completed the 9-item Patient Health Questionnaire (PHQ), a validated screen for depression [32]. Each question on this self-reported assessment of Diagnostic and Statistical Manual of Mental Disorders 4th edition signs and symptoms of depression is scored from 0 (not at all) to 3 (nearly every day), with a total possible score of 27 calculated by summing the scores of the nine individual questions. A total score of ten or higher is considered indicative of major depression [32].

### 2.4. Allostatic Load Biomarkers

The biomarkers included in this analysis as comprising the allostatic load (AL) are consistent with previous research [10,33,34]. These include three cardiovascular biomarkers (systolic and diastolic blood pressure (BP), and pulse rate); four metabolic markers (glycosolated hemoglobin, body mass index (BMI), high-density lipoprotein (HDL) cholesterol, and total cholesterol); and two immunological markers (serum albumin and c-reactive protein (CRP)). Systolic and diastolic BP values were calculated as the average of three readings. Biomarkers with values above the 75th percentile of nationally weighted empirical cutoffs were categorized as “high-risk”, with the exception of serum albumin and HDL cholesterol, which were categorized as “high-risk” for values below the 25th percentile empirical cutoff, as lower values of these biomarkers are considered indicative of poor physiological function. High-risk thresholds were as follows: systolic BP > 127.3 mmHG; diastolic BP > 76 mmHG; pulse rate > 82 bpm; glycosylated hemoglobin > 5.7%; BMI > 30.6; HDL cholesterol < 42 mg/dL; total cholesterol > 216 mg/dL; serum albumin < 4.1 g/dL; CRP > 0.37 mg/dL. Previous research indicates these cutoffs as the preferred method of calculating the components of AL [28,34,35]. To calculate total AL score, one point was assigned for each high-risk biomarker value, with a total possible score of 9. In accordance with the literature [10], we consider AL scores of 4 or higher as “high-risk”.

### 2.5. Covariates

In consideration of potential over-controlling for mediating variables, we strictly limited the covariates for which we adjusted [36]. We included age, family poverty-to-income ratio (PIR), and each biomarker as covariates in our primary analysis based on prior literature showing associations of age and socioeconomic status (SES) with both depression [22] and allostatic load [33]. Age was stratified into five groups (18–24, 25–34, 35–44, 45–54, and 55–64 years) across which AL is known to vary [22]. PIR is an index for the ratio of household income to the federal poverty level based on family size and state of residence. NHANES provides PIR for each participant [26]. We stratified our analysis into five categories of PIR—“At or below”, “>1 and ≤2×”, “>2 and ≤3×”, “>3 and ≤4×”, and “>4×” the federal poverty threshold—to better capture the distribution of the biomarkers and depression across socioeconomic status.

### 2.6. Statistical Analysis

Statistical analysis was conducted between 1 August 2016 and 15 October 2016. All analyses were weighted to represent black and white women and men nationally following National Center for Health Statistics guidelines. For univariate analyses, means or frequencies (%) were reported; Pearson’s chi-square was used to test for statistically significant differences of categorical variables. Four multivariable logistic models with a significance level of α = 0.05 estimated the odds of depression as a function of each biomarker stratified by gendered race, adjusting for age, PIR, and all other biomarkers. All analyses were conducted using STATA version 14 (StataCorp LLC, College Station, TX, USA); code is available in Supplementary Materials (Supplementary S1) [37].

## 3. Results

Exclusions resulted in an analytic sample of 6431 US adults, which represents approximately 113 million black and white women and men nationally. Sociodemographics, depression, and high-risk levels of each of the nine included biomarkers are reported in Table 1 by gendered race. Black persons were more likely to be hypertensive and women had higher pulse rates. Half of black women were obese, while the prevalence of obesity ranged from 30% to 34% in the other three groups. A greater percentage of black women also had low serum album (52%) and elevated CRP (45%) levels. At 35%, the prevalence of high levels of glyco-hemoglobin was highest among black men. White men had the highest prevalence of high total cholesterol (44%) and low HDL cholesterol (39%). With the exception of low HDL cholesterol, white women had the lowest prevalence of high-risk biomarker levels of all groups. Black persons and women were more likely to report elevated depressive symptoms.

The adjusted odds of depression associated with high-risk levels of each biomarker is reported in Table 2 stratified by gendered race. Adjusting for age, socioeconomic status, and all other biomarkers, high-risk CRP, serum album, and total cholesterol levels, as well as high-risk pulse rate were differentially associated with increased risk for depression across the four groups. High-risk CRP levels increased odds of depression among white women (aOR = 1.7, 95% CI: 1.1–2.6) and white men (aOR = 1.8, 95% CI: 1.1–2.8), while no statistically significant associations were found among black women (aOR = 0.8, 95% CI: 0.6–1.1) or black men (aOR = 0.9, 95% CI: 0.5–1.5). Similarly, adjusted odds ratios for high-risk pulse rates were 1.5 (95% CI: 1.1–2.2) and 1.8 (95% CI: 1.1–2.9) for white women and white men, respectively, but not statistically significant among black women (1.1, 95% CI: 0.7–1.6) or men (1.2, 95% CI: 0.6–2.4). Among black men only, high-risk levels of systolic BP (aOR = 1.7, 95% CI: 1.1–2.7) and serum albumin (aOR = 1.7, 95% CI: 1.0–2.9) predicted depression. High levels of total cholesterol were associated with depression among black women (aOR = 1.6, 95% CI: 1.0–2.7).

## 4. Discussion

Our results support the differential effect hypothesis. The relationship between a number of physiological markers of chronic stress and depressive symptoms varied with respect to gendered race. While the prevalence of depression and high-risk inflammation indicators were notably higher among black women than white women, black men, or white men, black women were the only of the four groups among whom inflammation was not associated with depression. The biomarkers associated with depression were also consistent among white women and men, but not among black persons. Adverse levels of serum album and systolic BP predicted depression in black men, while among black women, high-risk levels of total cholesterol were associated with depression.

### 4.1. Gendered Racial Variation in Manifestations of Chronic Stress

An extensive body of literature supports gender and racial differences in the prevalence of inflammation [37,38,39,40,41], as well as in the cardiovascular and metabolic biomarkers comprising the allostatic load [13,18,19,20]. Our findings are consistent with prior literature showing that the prevalence of elevated pulse rate and inflammation tends to be higher among women regardless of race [13,42]. Even accounting for socioeconomic position, black women are particularly susceptible to premature aging, chronic inflammation, and associated conditions such as obesity [10,20,33], results that are supported in this analysis. In line with extant research, we also found high levels of total cholesterol and low levels of HDL cholesterol to be more prevalent among men and white persons [43].

Data linking adverse social exposures to increased risk of inflammation and subsequently depression [13,43] provide some support for the differential effect hypothesis but fall short of accounting for the concomitant effects of gender and race on the experience of social stimuli and for how this interaction influences variability in risk for psychiatric disorder. In contrast, research grounded in intersectionality theory has identified gendered racial differences in the effects of stress on mental health, finding a stronger association between stressful life events and major depressive episodes among white men than black men while identifying no such interaction among women [44]. Another study examined how allostatic load differentially predicts depressive symptoms in black and white women and men, finding an association only among black men and white women [45]. In accordance with these findings, our results indicate different underlying disease relationships among black and white women and men, divergence in the predictors of depressive symptoms potentially steered by unmeasured psychosocial exposures that are unique to each gendered race. Such variability in the experience of stress and its psychiatric presentation may indicate the necessity for more nuanced approaches to patient evaluation and prescribing practices.

### 4.2. Divergence in the Pathways from Chronic Stress to Depression

As noted earlier, treatment efficacy among black and white women and men contributes to disparities in morbidity [6] and is likely complicated by differences in the neurobiological processes associated with depression between these groups. In this study, inflammatory markers predicted depression in all groups except black women. Specifically, CRP, elevated levels of which have been increasingly identified as a risk factor for depression [13,18], was associated with increased odds only among whites. Previous findings [27,28] have identified an association of CRP with depressive symptoms only among whites and not blacks. Our study further builds on this evidence by identifying within-race gender differences in the association of inflammation with depression, as well as in other gendered race group-specific markers of chronic stress predictive of depression which have been earlier noted in the literature [46]. These findings are of particular interest in light of other literature demonstrating symptom-specific associations of CRP with depression. In one study, investigators found that higher levels of inflammation are more likely to underlie depressive symptoms indicative of sickness behavior including fatigue, reduced appetite, withdrawal, and inhibited motivation [29]. As black persons are more likely to report these somatic symptoms, our findings stand in contrast to this evidence, providing further indication of distinct physiological pathways from stress associated with social inequity to the development of depression among black and white women and men.

These disparate relationships suggest that, while a genetic predisposition may contribute to the likelihood of developing risk factors for depression such as inflammation [42], the particular experience of the social environment that is predicated upon one’s gendered race plays an integral part in depression pathogenesis [47]. This assertion is consistent with evidence for an interactive effect of genotype and social context on depression that varies with gender [15]. Such social environmental exposures moderating the experience of stress and subsequent effects on mental health may include cultural influences on racially informed gender roles and expectations, as well as the frequency and severity of perceived prejudice [47,48,49,50].

Research suggests that white women are more likely to ruminate and self-blame following identity threat (e.g., following discrimination exposure) than black women or white men [4,47,48,49,50,51], a socialized stress response [49] that may contribute to a greater proclivity for affective symptoms of depression under certain kinds of chronic stress [48]. In contrast, coping with the co-occurrence of racial and gender identity-based stressors may have allowed for the development of a psychological fortitude that is protective against depression, or affective symptoms of depression, under these conditions among black women [52,53], despite greater exposure to genetic and psychosocial risk factors for inflammation. The chronic strain-inflammation pathway of depression may therefore be more applicable to those operating within sociocultural paradigms that yield psychological and behavioral responses to social status-based stress—responses that may in turn exacerbate biological vulnerability to depression. Given potentially diverging mechanisms, approaches to evaluating and developing treatment plans for patients presenting with depressive symptoms should consider the sociocultural factors associated with gendered race as drivers of differences in underlying disease causes.

### 4.3. Limitations

This study has important limitations requiring acknowledgement. While the PHQ-9 has been validated and shown to have strong reliability and validity within a range of racial and ethnic populations [53], and among women and men [54], the instrument assesses depression based on current symptoms. Depressed individuals being successfully treated with medication or therapy may not be captured by the PHQ-9. Accordingly, our estimates of the association between allostatic load biomarkers and depression may be underestimated, particularly among white women, who are most likely of the groups under study to seek and undergo treatment for depression [55,56,57,58]. Our analysis was limited to NHANES 2005–2010 because the 2005–2006 surveys were the first to include the PHQ-9, and 2009–2010 was the latest wave to include all nine biomarkers used to calculate AL. This limited sample size prevented further analysis of interactive effects within stratified models. Research examining these associations among larger and more contemporary cohorts of US adults is therefore needed.

The number of participants excluded for missing data could raise concern about the representativeness of our sample. However, among those excluded due to missing data on biomarkers, missingness was distributed independently such that excluding one biomarker would not recover a significant number of respondents. Similarly, family PIR missingness was approximately equally distributed across AL biomarkers, race, and gender. Among participants missing depression scores, approximately 24% were black and 36% white. This missingness by race was distributed approximately equally across men and women, although not across income categories; participants excluded for missing information on depression had lower family PIRs (data are not shown).

## 5. Conclusions

Our findings provide some evidence of fundamental differences in the underlying neurological processes leading to depression pathogenesis among US black and white women and men. Interventions designed to eradicate social inequities remain important to reduce racial and gender disparities in a number of chronic diseases. However, implementing such interventions are challenging due in part to sociopolitical barriers and a lack of consensus among policy makers for best practices. Additional approaches at the individual level may complement system-level efforts by targeting the specific pathways over which chronic, identity-based, psychosocial stressors act to cause depression in different sociodemographic groups. On the importance of causal frameworks in the treatment of psychiatric disorders, Aaron Lazare opined: “The complexity of the decision-making process resulting from the use of several models may unnecessarily limit the treatment options of the psychiatrist […] If the conceptual models and their use in clinical psychiatry are made explicit, a broader range of treatment modalities should be made available” [59].

This study raises important concerns about the efficacy of psychiatric treatments which neglect sociocultural influences on the presentation of both chronic stress and depressive symptoms. Refining drug and psychotherapies as appropriate for distinct depression etiologies may yield improved treatment outcomes and reduce disparities between black and white women and men. We also suggest that prevention efforts should further focus on building resilience that targets the specific vulnerabilities associated with an individual’s gendered race. Additional research exploring the psychosocial and cultural exposures that contribute to the varied manifestations of chronic stress and depression would significantly strengthen subsequent research investigating the nature of gendered racial differences in depression etiology.

## Figures and Tables

**Table 1:** Sample characteristics by gendered race in NHANES 2005–2010, weighted %.

Measures	Black Women	White Women	Black Men	White Men	*P* ^d^
Sample *N*	980	2147	1028	2276	
Weighted *N*	7,895,277	48,156,035	7,129,498	49,990,472	
Age, mean (SD)	40.7 (14.4)	41.7 (13.2)	40.2 (14.8)	41.4 (13.5)	
Family PIR ^a^					<0.001
≤1	25.3	10.7	19.6	8.3	
>1 and ≤2×	25.5	14.0	25.8	13.6	
>2 and ≤3×	15.6	13.4	18.4	13.9	
>3 and ≤4×	13.4	15.4	14.0	14.8	
>4	20.2	46.4	22.1	49.4	
High-risk AL Biomarkers ^b^					
Systolic BP	46.5	33.7	48.3	42.3	<0.001
Diastolic BP	50.3	40.4	50.7	50.2	<0.001
Pulse	25.9	24.0	13.5	16.0	<0.001
BMI	50.1	31.4	34.8	30.2	<0.001
Total cholesterol	36.4	43.7	36.3	44.4	<0.001
HDL cholesterol	12.5	14.3	27.3	39.3	<0.001
Glyco-hemoglobin	29.3	14.6	35.4	14.8	<0.001
Serum Albumin	52.4	29.8	23.1	10.5	<0.001
CRP	44.7	32.0	26.2	18.6	<0.001
High-risk AL	17.1	15.3	10.1	7.4	<0.001
Depression ^c^	14.6	8.6	7.1	4.9	<0.001

Abbreviations: AL = allostatic load; BP = blood pressure; BMI = body mass index; HDL = high-density lipoprotein; CRP = c-reactive protein; PIR = poverty-to-income ratio; ^a^ PIR is a ratio of household income to the US poverty threshold based on family size and state of residence; ^b^ “High-risk” thresholds for each biomarker were: systolic BP > 127.3 mmHG; diastolic BP > 76 mmHG; pulse rate > 82 bpm; glycosylated hemoglobin > 5.7%; BMI > 30.6; HDL cholesterol < 42 mg/dL; total cholesterol > 216 mg/dL; serum albumin < 4.1 g/dL; and CRP > 0.37 mg/dL; ^c^ PHQ-9 scores of ≥10; ^d^ *p*-value from Pearson’s chi-square test.

**Table 2:** Adjusted ^a^ odds of depression ^b^ with high-risk allostatic load and biomarker levels by gendered race in NHANES 2005–2010, OR (95% CI) ^c^.

Biomarker	Black Women	White Women	Black Men	White Men
Systolic BP	1.2 (0.6, 2.5)	1.1 (0.6, 2.0)	1.7 (1.1, 2.7) *	1.4 (0.8, 2.5)
Diastolic BP	1.1 (0.6, 2.1)	1.3 (0.8, 2.2)	1.2 (0.8, 1.9)	1.3 (0.8, 2.1)
Pulse	1.1 (0.7, 1.6)	1.5 (1.1, 2.2) *	1.2 (0.6, 2.4)	1.8 (1.1, 2.9) *
BMI	0.8 (0.5, 1.2)	1.1 (0.7, 1.7)	1.1 (0.6, 2.0)	0.9 (0.6, 1.3)
Total cholesterol	1.6 (1.0, 2.7) *	1.1 (0.8, 1.5)	1.0 (0.5, 2.0)	0.8 (0.4, 1.3)
HDL cholesterol	1.2 (0.6, 2.3)	1.1 (0.7, 1.7)	1.7 (0.9, 3.4)	1.3 (0.8, 1.9)
Glyco-hemoglobin	1.1 (0.8, 1.7)	1.0 (0.6, 1.7)	0.9 (0.5, 1.6)	0.8 (0.5, 1.4)
Serum Albumin	0.9 (0.6, 1.3)	1.0 (0.7, 1.6)	1.7 (1.0, 2.9) *	1.3 (0.7, 2.5)
CRP	0.8 (0.6, 1.1)	1.7 (1.1, 2.6) *	0.9 (0.5, 1.5)	1.8 (1.1, 2.8) *
High-risk AL ^d^	1.1 (0.6, 2.0)	2.1 (1.5, 3.0) *	1.7 (1.0, 2.9) *	1.4 (0.8, 2.5)

Abbreviations: BP = blood pressure; BMI = body mass index; HDL = high-density lipoprotein; CRP = c-reactive protein; ^a^ models adjusted for PIR (ratio of household income to the US poverty threshold), age, and all biomarkers; ^b^ PHQ-9 scores of ≥10; ^c^ results are from four separate regression models. The reference category for the biomarkers in each model is “low-risk”; ^d^ AL scores of ≥4 were considered “high-risk”.

## Supplementary Materials

The following are available online at http://www.mdpi.com/2227-9032/6/3/105/s1, Supplementary S1: stata code.
