# Supplementary material for: Allostatic Load Biomarker Associations with Depressive Symptoms Vary among US Black and White Women and Men
Source: Healthcare (Basel). 2018 Aug 28;6(3):105. doi: 10.3390/healthcare6030105 (PMC6163528; doi:10.3390/healthcare6030105)
Supplement: Supplementary file 1 [file healthcare-06-00105-s001.pdf]

Supplementary S1: Stata Code

```
***Recode and calculate variables for analysis of race/sex modification
of the the relationship between allostatic load and depression***set
more off
**Recode variables**
use "/Users/gangabey/Desktop/masterdataset_05-10.3.28.dta", clear

**Creating depression composite variable as binary yes/no -- final
version**
**Create new depression variable for each question
gen phq1=dpq01
gen phq2=dpq02
gen phq3=dpq03
gen phq4=dpq04
gen phq5=dpq05
gen phq6=dpq06
gen phq7=dpq07
gen phq8=dpq08
gen phq9=dpq09

**recode missing values**
replace phq1=.n if dpq010==9| dpq010 ==7
replace phq2=.n if dpq020==9| dpq020 ==7
replace phq3=.n if dpq030==9| dpq030 ==7
replace phq4=.n if dpq040==9| dpq040 ==7
replace phq5=.n if dpq050==9| dpq050 ==7
replace phq6=.n if dpq060==9| dpq060 ==7
replace phq7=.n if dpq070==9| dpq070 ==7
replace phq8=.n if dpq080==9| dpq080 ==7
replace phq9=.n if dpq090==9| dpq090 ==7

**Test
tab dpq01 phq1, missing
tab dpq02 phq2, missing
tab dpq03 phq3, missing
tab dpq04 phq4, missing
tab dpq05 phq5, missing
tab dpq06 phq6, missing
tab dpq07 phq7, missing
tab dpq08 phq8, missing
tab dpq09 phq9, missing

**Calculate composite PHQ9 score**
**Note that any participants missing any of 9 items will not get a total
score**
gen dpq_comp_GB = phq1 + phq2 + phq3 + phq4 + phq5 + phq6 + phq7 + phq8
+ phq9

**test
tabstat  dpq_comp_GB phq1 phq2 phq3 phq4 phq5 phq6 phq7 phq8 phq9,
stats(n) missing
**test for total scores even with missing components
list  dpq_comp_GB phq1 phq2 phq3 phq4 phq5 phq6 phq7 phq8 phq9 if
dpq_comp_GB !=. in 1/50
**test for score even if missing individual component (should not get
an output if coded correctly)
```

```
list   dpq_comp_GB  phq1  phq2  phq3  phq4  phq5  phq6  phq7  phq8  phq9  if
dpq_comp_GB !=. & (phq1==. | phq2==. | phq3==. | phq4==. | phq5==. |
phq6==. | phq7==. | phq8==. | phq9==.)

**Recode composite PHQ9 to binary, PHQ≥10 yes/no
gen dpq_compcat_GB =.
replace dpq_compcat_GB = 0 if (dpq_comp_GB <10) & dpq_comp_GB !=.
replace dpq_compcat_GB = 1 if (dpq_comp_GB >=10) & (dpq_comp_GB <28) &
dpq_comp_GB !=.
label define dpq_compcat_GB1 0 "0.No" 1 "1.Yes"
label values dpq_compcat_GB dpq_compcat_GB1
label variable dpq_compcat_GB "PHQ9>=10"

**test
tabstat dpq_comp_GB, by(dpq_compcat_GB) stats (n min max) missing

**Creating Allostatic Load composite variable**

**Create new variable for AL composite score using empirical cutoff
values for each variable (+1 pt) including:

**systolic blood press - bpxsy  >75th %
**diastolic blood pressure - bpxdi  >75th %
**BMI - bmxbmi >75th %
**c-reactive protein - lbxcrp <25th %
**glycosolated hemoglobin - lbxgh >75th %
**HDL cholesterol - lbdhdd <25th %
**albumin - lbxsal >75th %
**total cholesterol - lbxtc >75th %
**pulse rate - bpxpls >75th %

***Create systolic and diastolic score variable***
**create new variable for systolic and diastolic blood pressure average
of 3 readings**
**NOTE: Avg will only be calculated for participants with 3 readings
*systolic*

gen nhave_bpxsy = 0
replace nhave_bpxsy = nhave_bpxsy + 1 if bpxsy1 !=.
replace nhave_bpxsy = nhave_bpxsy + 1 if bpxsy2 !=.
replace nhave_bpxsy = nhave_bpxsy + 1 if bpxsy3 !=.
replace nhave_bpxsy = nhave_bpxsy + 1 if bpxsy4 !=.

gen tot_bpxsy = 0
replace tot_bpxsy = tot_bpxsy + bpxsy1 if bpxsy1 !=.
replace tot_bpxsy = tot_bpxsy + bpxsy2 if bpxsy2 !=.
replace tot_bpxsy = tot_bpxsy + bpxsy3 if bpxsy3 !=.
replace tot_bpxsy = tot_bpxsy + bpxsy4 if bpxsy4 !=.
replace tot_bpxsy = . if nhave_bpxsy != 3

gen bpxsy_avg = tot_bpxsy/3

**test...?
tabstat bpxsy_avg, by(nhave_bpxsy) stats(n) missing
**test...?
tabstat bpxsy_avg bpxsy1 bpxsy2 bpxsy3 bpxsy4, by(nhave_bpxsy) stats(n)
missing
**test that bpxsy_avg is actually average of 3 BP readings
list bpxsy_avg bpxsy1 bpxsy2 bpxsy3 bpxsy4 nhave_bpxsy in 1/50
```

```
*diastolic*
gen nhave_bpxdi = 0
replace nhave_bpxdi = nhave_bpxdi + 1 if bpxdi1 !=.
replace nhave_bpxdi = nhave_bpxdi + 1 if bpxdi2 !=.
replace nhave_bpxdi = nhave_bpxdi + 1 if bpxdi3 !=.
replace nhave_bpxdi = nhave_bpxdi + 1 if bpxdi4 !=.

gen tot_bpxdi = 0
replace tot_bpxdi = tot_bpxdi + bpxdi1 if bpxdi1 !=.
replace tot_bpxdi = tot_bpxdi + bpxdi2 if bpxdi2 !=.
replace tot_bpxdi = tot_bpxdi + bpxdi3 if bpxdi3 !=.
replace tot_bpxdi = tot_bpxdi + bpxdi4 if bpxdi4 !=.
replace tot_bpxdi = . if nhave_bpxdi != 3

gen bpxdi_avg = tot_bpxdi/3

**test
tabstat bpxdi_avg, by(nhave_bpxdi) stats(n) missing
**test...?
tabstat bpxdi_avg bpxdi1 bpxdi2 bpxdi3 bpxdi4, by(nhave_bpxdi) stats(n)
missing
**test that bpxdi_avg is actually average of 3 BP readings
list bpxdi_avg bpxdi1 bpxdi2 bpxdi3 bpxdi4 nhave_bpxdi in 1/50

**create systolic and diastolic blood pressure percentile variable**
* 1 systolic (or yes to having high blood pressure)*
**bpxsy_avg 75th % = 127.3

gen bpxsy_avg_compscore =.
replace bpxsy_avg_compscore = 0 if bpxsy_avg <= 127.3 & bpxsy_avg !=.
replace bpxsy_avg_compscore = 1 if bpxsy_avg > 127.3 & bpxsy_avg !=.
replace bpxsy_avg_compscore = 1 if bpq020==1

**test
tabstat bpxsy_avg, by(bpxsy_avg_compscore) stats(n min max) missing
**test that all participants with high BP diagnosis have been assigned
1 in bpxsy_avg_compscore
tab bpq020, missing
tab bpq020 bpxsy_avg_compscore, missing
**test that participants assigned 1 in bpxsy_avg_compscore even with BP
avg below 127.3 all answered yes to dpq020
tab bpq020 bpxsy_avg_compscore if bpxsy_avg_compscore==1 & bpxsy_avg
<127.3
tabstat bpxsy_avg if bpq020==1, by(bpxsy_avg_compscore) stats(n min
max) missing
tabstat bpxsy_avg if bpq020!=1, by(bpxsy_avg_compscore) stats(n min
max) missing
**test that participants without 3 BP readings or diagnosed with high
BP are not assigned 1 in bpxsy_avg_compscore (should not get output if
coded correctly)
list bpq020 bpxsy_avg bpxsy_avg bpxsy1 bpxsy2 bpxsy3 bpxsy4 nhave_bpxsy
if bpq020==1 & bpxsy_avg_compscore==.
tab nhave_bpxsy if bpq020==1 & bpxsy_avg_compscore==.

* 2 diastolic*
**bpxdi_avg 75th % = 76

gen bpxdi_avg_compscore =.
replace bpxdi_avg_compscore = 0 if bpxdi_avg <= 76 & bpxdi_avg !=.
replace bpxdi_avg_compscore = 1 if bpxdi_avg > 76 & bpxdi_avg !=.
```

```
replace bpxdi_avg_compscore = 1 if bpq020==1

**test
tabstat bpxdi_avg, by(bpxdi_avg_compscore) stats(n min max) missing
**test that all participants with high BP diagnosis have been assigned
1 in bpxdi_avg_compscore
tab bpq020, missing
tab bpq020 bpxdi_avg_compscore, missing
**test that participants assigned 1 in bpxdi_avg_compscore even with BP
avg below 76 all answered yes to bpq020
tab bpq020 bpxdi_avg_compscore if bpxdi_avg_compscore==1 & bpxdi_avg
<76
tabstat bpxdi_avg if bpq020==1, by(bpxdi_avg_compscore) stats(n min
max) missing
tabstat bpxdi_avg if bpq020!=1, by(bpxdi_avg_compscore) stats(n min
max) missing
**test that pts w/o 3 BP readings or high BP diagnosis are not assigned
1 in bpxdi_avg_compscore (should not get output if coded correctly)
list bpq020 bpxdi_avg bpxdi_avg bpxdi1 bpxdi2 bpxdi3 bpxdi4 nhave_bpxdi
if bpq020==1 & bpxdi_avg_compscore==.
tab nhave_bpxdi if bpq020==1 & bpxdi_avg_compscore==.

***3 BMI score variable***
gen bmxbmi_compscore =.
replace bmxbmi_compscore = 0 if bmxbmi <= 30.6 & bmxbmi !=.
replace bmxbmi_compscore = 1 if bmxbmi > 30.6 & bmxbmi !=.

**test
tabstat bmxbmi, by(bmxbmi_compscore) stats(n min max) missing

***4 c-reactive protein score variable***
gen lbxcrp_compscore =.
replace lbxcrp_compscore = 0 if lbxcrp <= .37 & lbxcrp !=.
replace lbxcrp_compscore = 1 if lbxcrp > .37 & lbxcrp !=.

**test
tabstat lbxcrp, by(lbxcrp_compscore) stats(n min max) missing

***5 glycosolated hemoglobin score variable (or yes to having
diabetes)***
gen lbxgh_compscore =.
replace lbxgh_compscore = 0 if lbxgh <= 5.7 & lbxgh !=.
replace lbxgh_compscore = 1 if lbxgh > 5.7 & lbxgh !=.
replace lbxgh_compscore = 1 if diq010 ==1

**test
tabstat lbxgh, by(lbxgh_compscore) stats(n min max) missing
**test that all participants with diabetes diagnosis have been assigned
1 in lbxgh_compscore
tab diq010, missing
tab diq010 lbxgh_compscore, missing
**test that participants assigned 1 in lbxgh_compscore even with glyc
hem below 5.7 all answered yes to diq010
tab diq010 lbxgh_compscore if lbxgh_compscore==1 & lbxgh <5.7
tabstat lbxgh if diq010==1, by(lbxgh_compscore) stats(n min max) missing

tabstat lbxgh if diq010!=1, by(lbxgh_compscore) stats(n min max) missing
**test that pts w/o gly hem value or diabetes diagnosis are not assigned
1 in lbxgh_compscore (should not get output if coded correctly)
list diq010 lbxgh if diq010==1 & lbxgh_compscore==.
```

```
***6 HDL cholesterol score variable***
gen lbdhdd_compscore =.
replace lbdhdd_compscore = 0 if lbdhdd >= 42 & lbdhdd !=.
replace lbdhdd_compscore = 1 if lbdhdd < 42 & lbdhdd !=.

**test
tabstat lbdhdd, by(lbdhdd_compscore) stats(n min max) missing

***7 albumin score variable***
gen lbxsal_compscore =.
replace lbxsal_compscore = 0 if lbxsal >= 4.1 & lbxsal !=.
replace lbxsal_compscore = 1 if lbxsal < 4.1 & lbxsal !=.

**test
tabstat lbxsal, by(lbxsal_compscore) stats(n min max) missing

***8 total cholesterol score variable (or yes to having high
cholesterol)***
gen lbxtc_compscore =.
replace lbxtc_compscore = 0 if lbxtc <= 216 & lbxtc !=.
replace lbxtc_compscore = 1 if lbxtc > 216 & lbxtc !=.
replace lbxtc_compscore = 1 if bpq080==1

**test
tabstat lbxtc, by(lbxtc_compscore) stats(n min max) missing
**test that all participants with high chol diagnosis have been assigned
1 in lbxtc_compscore
tab bpq080, missing
tab bpq080 lbxtc_compscore, missing
**test that participants assigned 1 in lbxtc_compscore even with tot
chol below 216 all answered yes to bpq080
tab bpq080 bpxdi_avg_compscore if lbxtc_compscore==1 & lbxtc <216
tabstat lbxtc if bpq080==1, by(lbxtc_compscore) stats(n min max) missing

tabstat lbxtc if bpq080!=1, by(lbxtc_compscore) stats(n min max) missing
**test that pts w/o tot chol values or high chol diagnosis are not
assigned 1 in lbxtc_compscore (should not get output if coded correctly)
list bpq080 lbxtc  if bpq080==1 & lbxtc_compscore==.

***9 pulse rate score variable***
gen bpxpls_compscore =.
replace bpxpls_compscore = 0 if bpxpls <= 82 & bpxpls !=.
replace bpxpls_compscore = 1 if bpxpls > 82 & bpxpls !=.

**test
tabstat bpxpls, by(bpxpls_compscore) stats(n min max) missing

***Allostatic Load composite score variable.***
**Note that participants missing any of the 9 components will not get
a total score**
gen AL_compscore = bpxsy_avg_compscore + bpxdi_avg_compscore +
bmxbmi_compscore + lbxcrp_compscore + lbxgh_compscore +
lbdhdd_compscore + lbxsal_compscore + lbxtc_compscore +
bpxpls_compscore

**test
tabstat AL_compscore bpxsy_avg_compscore bpxdi_avg_compscore
lbxcrp_compscore lbxgh_compscore lbdhdd_compscore lbxsal_compscore
lbxtc_compscore bpxpls_compscore, stats(n) missing
```

```
**test for total scores even with missing components
list AL_compscore bpxsy_avg_compscore bpxdi_avg_compscore lbxcrp_compscore lbxgh_compscore lbdhdd_compscore lbxsal_compscore lbxtc_compscore bpxpls_compscore if AL_compscore !=. in 1/5
**test for score even if missing individual component (should not get an output if coded correctly)
list AL_compscore bpxsy_avg_compscore bpxdi_avg_compscore lbxcrp_compscore lbxgh_compscore lbdhdd_compscore lbxsal_compscore lbxtc_compscore bpxpls_compscore if AL_compscore !=. & (bpxsy_avg_compscore==.| bpxdi_avg_compscore==.| lbxcrp_compscore==.| lbxgh_compscore==.| lbdhdd_compscore==.| lbxsal_compscore==.| lbxtc_compscore==.| bpxpls_compscore==.)

*Allostatic load as a binary variable*
gen AL_risk=.
replace AL_risk =0 if AL_compscore <= 4 & AL_compscore !=.
replace AL_risk =1 if AL_compscore > 4 & AL_compscore !=.
label define AL_risk1 0 "0.Low-risk" 1 "1.High-risk"
label values AL_risk AL_risk1

**test
tabstat AL_compscore, by(AL_risk) stats(n min max) missing

**Creating interaction and confounding variables**

**Race/Sex**
gen FEMALE=.
replace FEMALE = 0 if (riagendr==1)
replace FEMALE = 1 if (riagendr==2)

label define FEMALE1 0 "0.Male" 1 "1.Female"
label values FEMALE FEMALE1

**test
tab FEMALE riagendr, missing

**race variable, limit to black and white participants**
gen RACE_BLACK=.
replace RACE_BLACK = 0 if (ridreth1==3)
replace RACE_BLACK = 1 if (ridreth1==4)

label define RACE_BLACK1 0 "0.Non-Hispanic White" 1 "1.Non-Hispanic Black"

label values RACE_BLACK RACE_BLACK1

**test
tab ridreth1
tab RACE_BLACK ridreth1, missing

**Creating race/sex variable for interaction with allostatic load composite variable**
gen race_sex=.
replace race_sex = 4 if FEMALE==1 & RACE_BLACK==1
replace race_sex = 3 if FEMALE==0 & RACE_BLACK==1
replace race_sex = 2 if FEMALE==1 & RACE_BLACK==0
replace race_sex = 1 if FEMALE==0 & RACE_BLACK==0

label define race_sex1 1 "1.White male" 2 "2.White female" 3 "3.Black male" 4 "4.Black female"
```

```
label values race_sex race_sex1

**test
tab ridreth1
tab race_sex RACE_BLACK, missing
tab race_sex FEMALE, missing
tab FEMALE RACE_BLACK if race_sex==., missing
tab FEMALE RACE_BLACK if race_sex==1, missing
tab FEMALE RACE_BLACK if race_sex==2, missing
tab FEMALE RACE_BLACK if race_sex==3, missing
tab FEMALE RACE_BLACK if race_sex==4, missing


**Family PIR**
**Recoding and labeling Family PIR variable**
gen pir_GB=.
replace pir_GB = 5 if indfmpir <= 1 & indfmpir !=.
replace pir_GB = 4 if indfmpir > 1 & indfmpir <= 2 & indfmpir !=.
replace pir_GB = 3 if indfmpir > 2 & indfmpir <= 3 & indfmpir !=.
replace pir_GB = 2 if indfmpir > 3 & indfmpir <= 4 & indfmpir !=.
replace pir_GB = 1 if indfmpir > 4 & indfmpir !=.

label define pir_GB1 5 "At or below poverty" 4 ">1 and ≤2 times poverty"
3 ">2 and ≤3 times poverty" 2 ">3 and ≤4 times poverty" 1 ">4 times
poverty"

label values pir_GB pir_GB1

**test
tabstat indfmpir, statistics (count min max) by(pir_GB) missing

**Age**
**Recoding and labeling age variable**
gen age_GB=.
replace age_GB = 1 if ridageyr >= 18 & ridageyr <= 24
replace age_GB = 2 if ridageyr >= 25 & ridageyr <= 34
replace age_GB = 3 if ridageyr >= 35 & ridageyr <= 44
replace age_GB = 4 if ridageyr >= 45 & ridageyr <= 54
replace age_GB = 5 if ridageyr >= 55 & ridageyr <= 64
label define age_GB1 1 "18-24" 2 "25-34" 3 "35-44" 4 "45-54" 5 "55-64"
label values age_GB age_GB1
label variable age_GB "Age categories: 18-64yo only"

**test
tabstat ridageyr, statistics (count min max) by(age_GB) missing
tabstat ridageyr if ridageyr<20, statistics (count min max) by(age_GB)
missing
tabstat ridageyr if ridageyr>63, statistics (count min max) by(age_GB)
missing

*** Create eligibility variable
* Exclude if <18yo, >64yo, race does not equal black or white, missing
info on age, race, gender, family PIR,
*depression, medication use, pulse, bmi, CRP, glyco-Hb, HDL chol, Tchol
albumin, diabetes diagnosis, BP and Chol diagnosis
generate myelig = 1
**missing age
replace myelig = 0 if age_GB == .
**out of age range
```

```
replace myelig = 0 if ridageyr <18 | ridageyr >64
**missing race, or not black or white
replace myelig = 0 if RACE_BLACK == .
**missing gender
replace myelig = 0 if FEMALE == .
**missing PHQ9 depression composite score
replace myelig = 0 if dpq_compcat_GB == .
**missing family PIR
replace myelig = 0 if pir_GB == .
**missing AL composite score
replace myelig = 0 if AL_risk == .
**pregnant women
replace myelig = 0 if ridexprg == 1

label define eligf 0 "0.ineligible" 1 "1.eligible"
label values myelig eligf
label variable myelig "Analytic sample"

**test
tabstat dpq_compcat_GB AL_compscore age_GB RACE_BLACK FEMALE pir_GB,
by(myelig) stats(n min max) missing
**test: age exclusion
gen ageOUT = 1
replace ageOUT = 0 if age_GB ==.
label define ageOUT 1 "include" 0 "exclude"
label values ageOUT ageOUTf
tab myelig ageOUT, missing
tabstat ridageyr, by(ageOUT) stats (n min max) missing
tab ridageyr if ageOUT==1
**test: missing depression score exclusion
gen dpqOUT = 1
replace dpqOUT = 0 if dpq_compcat_GB ==.
label define dpqOUT 1 "include" 0 "exclude"
label values dpqOUT dpqOUTf
tab myelig dpqOUT, missing
**test: missing AL score exclusion
gen ALscoreOUT = 1
replace ALscoreOUT = 0 if AL_compscore ==.
label define ALscoreOUT 1 "include" 0 "exclude"
label values ALscoreOUT ALscoreOUTf
tab myelig ALscoreOUT, missing
**test: missing PIR info exclusion
gen pirOUT = 1
replace pirOUT = 0 if pir_GB ==.
label define pirOUT 1 "include" 0 "exclude"
label values pirOUT pirOUTf
tab myelig pirOUT, missing
**test: missing race or gender exclusion
gen race_sexOUT = 1
replace race_sexOUT = 0 if race_sex ==.
label define race_sexOUT 1 "include" 0 "exclude"
label values race_sexOUT race_sexOUTf
tab myelig race_sexOUT, missing

**apply sample weights (6yr, 3 wave)
generate mymecwt6yr = wtmec2yr * 1/3
svyset sdmvpsu [pweight=mymecwt6yr], strata(sdmvstra) vce(linearized)

**test
svy, vce(linearized): mean ridageyr
```

```
***Subgroup analysis***
**Black subjects**
gen myelig_black = 0
replace myelig_black = 1 if myelig==1 & RACE_BLACK==1

**test
tab myelig_black myelig, missing
tab myelig_black RACE_BLACK, missing
tab myelig RACE_BLACK if myelig_black==.
tab myelig RACE_BLACK if myelig_black==0
tab myelig RACE_BLACK if myelig_black==1

**White subjects**
gen myelig_white = 0
replace myelig_white = 1 if myelig==1 & RACE_BLACK==0

**test
tab myelig_white myelig, missing
tab myelig_white RACE_BLACK, missing
tab myelig RACE_BLACK if myelig_white==.
tab myelig RACE_BLACK if myelig_white==0
tab myelig RACE_BLACK if myelig_white==1

**Female subjects**
gen myelig_female = 0
replace myelig_female = 1 if myelig==1 & FEMALE==1

**test
tab myelig_female myelig, missing
tab myelig_female FEMALE, missing
tab myelig FEMALE if myelig_female==.
tab myelig FEMALE if myelig_female==0
tab myelig FEMALE if myelig_female==1

**Male subjects**
gen myelig_male = 0
replace myelig_male = 1 if myelig==1 & FEMALE==0

**test
tab myelig_male myelig, missing
tab myelig_male FEMALE, missing
tab myelig FEMALE if myelig_male==.
tab myelig FEMALE if myelig_male==0
tab myelig FEMALE if myelig_male==1

**Race/gender subgroups**
*Black female
gen myelig_bf = 0
replace myelig_bf = 1 if myelig==1 & FEMALE==1 & RACE_BLACK==1

**test
tab myelig_bf myelig, missing
tab myelig_bf FEMALE, missing
tab myelig_bf RACE_BLACK, missing
tab myelig FEMALE if myelig_bf==.
tab myelig RACE_BLACK if myelig_bf==.
tab myelig FEMALE if myelig_bf==1
tab myelig RACE_BLACK if myelig_bf==1
```

```
*Black male
gen myelig_bm = 0
replace myelig_bm = 1 if myelig==1 & FEMALE==0 & RACE_BLACK==1

**test
tab myelig_bm myelig, missing
tab myelig_bm FEMALE, missing
tab myelig_bm RACE_BLACK, missing
tab myelig FEMALE if myelig_bm==.
tab myelig RACE_BLACK if myelig_bm==.
tab myelig FEMALE if myelig_bm==1
tab myelig RACE_BLACK if myelig_bm==1

*White female
gen myelig_wf = 0
replace myelig_wf = 1 if myelig==1 & FEMALE==1 & RACE_BLACK==0

**test
tab myelig_wf myelig, missing
tab myelig_wf FEMALE, missing
tab myelig_wf RACE_BLACK, missing
tab myelig FEMALE if myelig_wf==.
tab myelig RACE_BLACK if myelig_wf==.
tab myelig FEMALE if myelig_wf==1
tab myelig RACE_BLACK if myelig_wf==1

*White male
gen myelig_wm = 0
replace myelig_wm = 1 if myelig==1 & FEMALE==0 & RACE_BLACK==0

**test
tab myelig_wm myelig, missing
tab myelig_wm FEMALE, missing
tab myelig_wm RACE_BLACK, missing
tab myelig FEMALE if myelig_wm==.
tab myelig RACE_BLACK if myelig_wm==.
tab myelig FEMALE if myelig_wm==1
tab myelig RACE_BLACK if myelig_wm==1

*eligible by gendered race
gen myelig_gnrdrc =.
replace myelig_gnrdrc=1 if myelig==1 & myelig_bf==1
replace myelig_gnrdrc=2 if myelig==1 & myelig_bm==1
replace myelig_gnrdrc=3 if myelig==1 & myelig_wf==1
replace myelig_gnrdrc=4 if myelig==1 & myelig_wm==1

*test
tab myelig_gnrdrc race_sex

***Numbers in manuscript***

**Exclusion criteria**
**age
tab age_GB, missing
**Race
tabstat age_GB, by(RACE_BLACK) stats (n min max) missing
**PHQ-9 data
tabstat age_GB, by(dpq_compcat_GB) stats (n min max) missing
**AL score
tabstat age_GB, by(AL_risk) stats (n min max) missing
```

```
**Family PIR
tabstat age_GB, by(pir_GB) stats (n min max) missing
**Pregnancy
tabstat age_GB, by(ridexprg) stats (n min max) missing
**Total ineligible
tab myelig
**Representive population size
svy, subpop(myelig) vce(linearized): mean AL_compscore

*Table 1: Proprotion high-risk by race-sex group
**Blood pressure
*Systolic
svy, subpop(myelig) vce(linearized): proportion bpxsy_avg_compscore
svy, subpop(myelig_bf) vce(linearized): proportion bpxsy_avg_compscore
svy, subpop(myelig_bm) vce(linearized): proportion bpxsy_avg_compscore
svy, subpop(myelig_wf) vce(linearized): proportion bpxsy_avg_compscore
svy, subpop(myelig_wm) vce(linearized): proportion bpxsy_avg_compscore

*Diastolic
svy, subpop(myelig) vce(linearized): proportion bpxdi_avg_compscore
svy, subpop(myelig_bf) vce(linearized): proportion bpxdi_avg_compscore
svy, subpop(myelig_bm) vce(linearized): proportion bpxdi_avg_compscore
svy, subpop(myelig_wf) vce(linearized): proportion bpxdi_avg_compscore
svy, subpop(myelig_wm) vce(linearized): proportion bpxdi_avg_compscore

**Pulse rate
svy, subpop(myelig) vce(linearized): proportion bpxpls_compscore
svy, subpop(myelig_bf) vce(linearized): proportion bpxpls_compscore
svy, subpop(myelig_bm) vce(linearized): proportion bpxpls_compscore
svy, subpop(myelig_wf) vce(linearized): proportion bpxpls_compscore
svy, subpop(myelig_wm) vce(linearized): proportion bpxpls_compscore

**BMI
svy, subpop(myelig) vce(linearized): proportion bmxbmi_compscore
svy, subpop(myelig_bf) vce(linearized): proportion bmxbmi_compscore
svy, subpop(myelig_bm) vce(linearized): proportion bmxbmi_compscore
svy, subpop(myelig_wf) vce(linearized): proportion bmxbmi_compscore
svy, subpop(myelig_wm) vce(linearized): proportion bmxbmi_compscore

**C-reactive protein
svy, subpop(myelig) vce(linearized): proportion lbxcrp_compscore
svy, subpop(myelig_bf) vce(linearized): proportion lbxcrp_compscore
svy, subpop(myelig_bm) vce(linearized): proportion lbxcrp_compscore
svy, subpop(myelig_wf) vce(linearized): proportion lbxcrp_compscore
svy, subpop(myelig_wm) vce(linearized): proportion lbxcrp_compscore

**Glycosolated Hemoglobin
svy, subpop(myelig) vce(linearized): proportion lbxgh_compscore
svy, subpop(myelig_bf) vce(linearized): proportion lbxgh_compscore
svy, subpop(myelig_bm) vce(linearized): proportion lbxgh_compscore
svy, subpop(myelig_wf) vce(linearized): proportion lbxgh_compscore
svy, subpop(myelig_wm) vce(linearized): proportion lbxgh_compscore

**HDL Cholesterol
svy, subpop(myelig) vce(linearized): proportion lbdhdd_compscore
svy, subpop(myelig_bf) vce(linearized): proportion lbdhdd_compscore
svy, subpop(myelig_bm) vce(linearized): proportion lbdhdd_compscore
svy, subpop(myelig_wf) vce(linearized): proportion lbdhdd_compscore
svy, subpop(myelig_wm) vce(linearized): proportion lbdhdd_compscore
```

```
**Total Cholesterol
svy, subpop(myelig) vce(linearized): proportion lbxtc_compscore
svy, subpop(myelig_bf) vce(linearized): proportion lbxtc_compscore
svy, subpop(myelig_bm) vce(linearized): proportion lbxtc_compscore
svy, subpop(myelig_wf) vce(linearized): proportion lbxtc_compscore
svy, subpop(myelig_wm) vce(linearized): proportion lbxtc_compscore

**Serum Albumin
svy, subpop(myelig) vce(linearized): proportion lbxsal_compscore
svy, subpop(myelig_bf) vce(linearized): proportion lbxsal_compscore
svy, subpop(myelig_bm) vce(linearized): proportion lbxsal_compscore
svy, subpop(myelig_wf) vce(linearized): proportion lbxsal_compscore
svy, subpop(myelig_wm) vce(linearized): proportion lbxsal_compscore

*Table 2: Adjusted Odds of Depression by AL Biomarker and Race/Sex
group, OR (95% CI)*
svy, subpop(myelig) vce(linearized): logistic dpq_compcat pir_GB age_GB
bpxsy_avg_compscore bpxdi_avg_compscore bpxpls_compscore
bmxbmi_compscore lbxtc_compscore lbdhdd_compscore lbxsal_compscore
lbxcrp_compscore lbxgh_compscore
svy, subpop(myelig_bf) vce(linearized): logistic dpq_compcat pir_GB
age_GB bpxsy_avg_compscore bpxdi_avg_compscore bpxpls_compscore
bmxbmi_compscore lbxtc_compscore lbdhdd_compscore lbxsal_compscore
lbxcrp_compscore lbxgh_compscore
svy, subpop(myelig_bm) vce(linearized): logistic dpq_compcat pir_GB
age_GB bpxsy_avg_compscore bpxdi_avg_compscore bpxpls_compscore
bmxbmi_compscore lbxtc_compscore lbdhdd_compscore lbxsal_compscore
lbxcrp_compscore lbxgh_compscore
svy, subpop(myelig_wf) vce(linearized): logistic dpq_compcat pir_GB
age_GB bpxsy_avg_compscore bpxdi_avg_compscore bpxpls_compscore
bmxbmi_compscore lbxtc_compscore lbdhdd_compscore lbxsal_compscore
lbxcrp_compscore lbxgh_compscore
svy, subpop(myelig_wm) vce(linearized): logistic dpq_compcat pir_GB
age_GB bpxsy_avg_compscore bpxdi_avg_compscore bpxpls_compscore
bmxbmi_compscore lbxtc_compscore lbdhdd_compscore lbxsal_compscore
lbxcrp_compscore lbxgh_compscore

*Missingness analysis*

**missing biomarkers

*number (of 14,050 black and white participants) missing at least 1/10
PHQ9 answers
tabstat age_GB, by (dpq_comp_GB) stats(n) missing
*1824*

*number (of 14,050 black and white participants) missing at least 2/4
systolic bp readings
tabstat age_GB, by (bpxsy_avg_compscore) stats(n) missing
*1492*

*number (of 14,050 black and white participants) missing at least 2/4
diastolic bp readings
tabstat age_GB, by (bpxdi_avg_compscore) stats(n) missing
*1492*

*number (of 14,050 black and white participants) missing bmi
tabstat age_GB, by (bmxbmi_compscore) stats(n) missing
*562*
```

```
*number (of 14,050 black and white participants) missing crp
tabstat age_GB, by (lbxcrp_compscore) stats(n) missing
*1247*

*number (of 14,050 black and white participants) missing glycohemoglobin
tabstat age_GB, by (lbxgh_compscore) stats(n) missing
*1127*

*number (of 14,050 black and white participants) missing HDL cholesterol
tabstat age_GB, by (lbdhdd_compscore) stats(n) missing
*1284*

*number (of 14,050 black and white participants) missing serum albumin
tabstat age_GB, by (lbxsal_compscore) stats(n) missing
*1320*

*number (of 14,050 black and white participants) missing total
cholesterol
tabstat age_GB, by (lbxtc_compscore) stats(n) missing
*1058*

*number (of 14,050 black and white participants) missing pulse rate
tabstat age_GB, by (bpxpls_compscore) stats(n) missing
*1038*

**missing PIR

*missing PIR by race
tab pir_GB RACE_BLACK, chi2 missing
*~6% missing among whites, 7% missing among blacks

*missing PIR by gender
tab pir_GB FEMALE, chi2 missing
*~7% missing among men, 8% missing among women

*missing PIR by AL_risk
tab pir_GB AL_risk, chi2 missing
*~7% missing among low-risk, 8% missing among high-risk

**missing depression score

gen black_miss_dpq =.
replace black_miss_dpq = 1 if RACE_BLACK==1 & dpq_compcat_GB ==. &
age_GB !=.
replace black_miss_dpq= 0 if RACE_BLACK==0 & dpq_compcat_GB==. & age_GB
!=.
tab dpq_compcat_GB black_miss_dpq, missing
tab black_miss_dpq FEMALE, chi2
tab black_miss_dpq pir_GB, chi2

*missing depression score by gender
tab dpq_compcat_GB FEMALE, chi2 missing
*~50% missing among men, 50% missing among blacks

*missing depression score by AL_risk
tab pir_GB AL_risk, chi2 missing
*~7% missing among low-risk, 8% missing among high-risk
```
